# Supplementary figures and images for: Sites of vulnerability on ricin B chain revealed through epitope mapping of toxin-neutralizing monoclonal antibodies
Source: PLoS One. 2020 Nov 9;15(11):e0236538. doi: 10.1371/journal.pone.0236538 (PMC7652295; doi:10.1371/journal.pone.0236538)

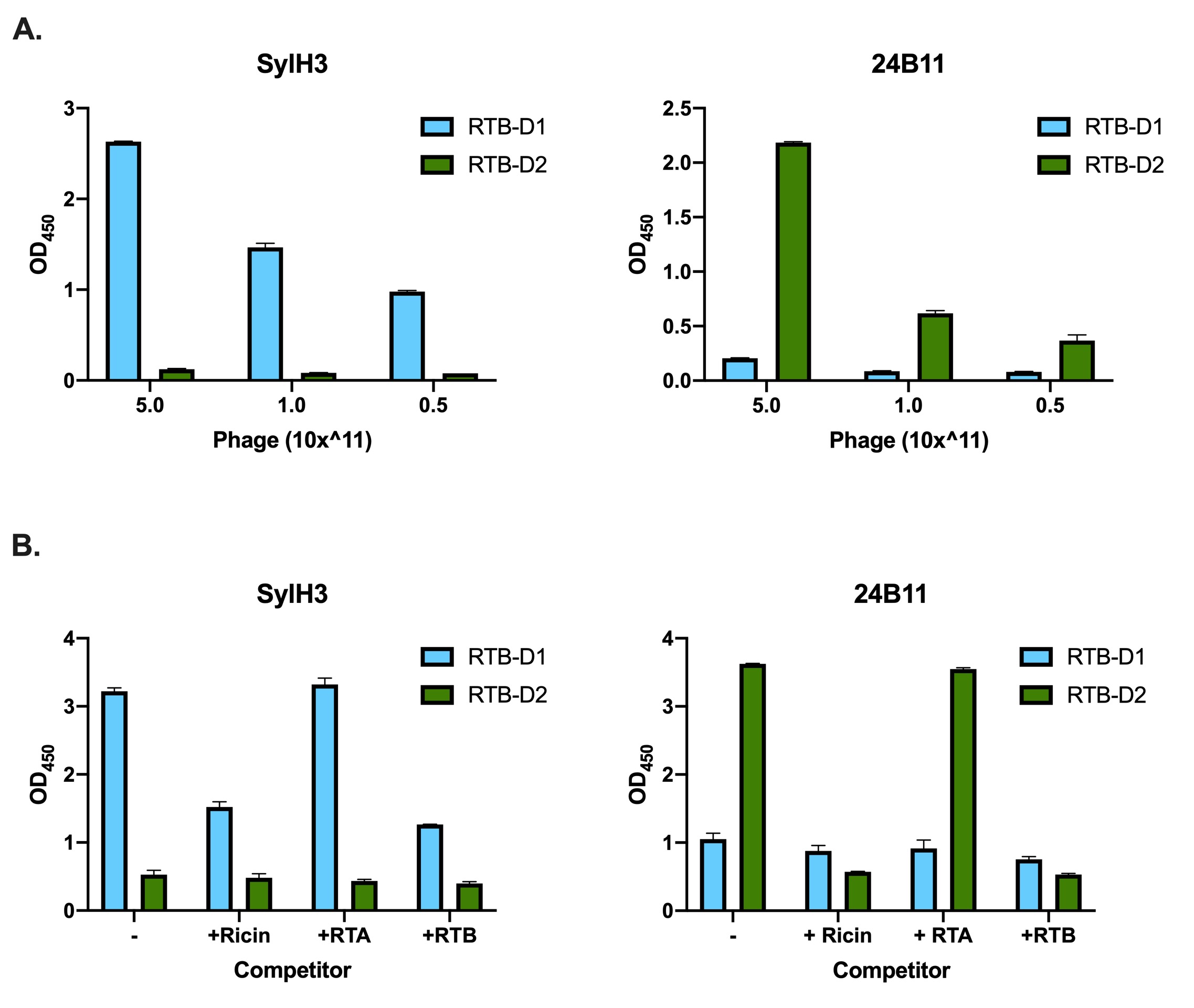

Supplement: S1 Fig — (A) Dose-dependent capture of RTB-D1 and RTB-D2 by plate-bound SylH3 and 24B11. As described in the Material and Methods, microtiter plates were coated with indicated mAbs (1 μg/mL in PBS) then probed with indicated number of RTB-D1 or RTB-D2 plaque forming units (PFU) per mL. The plates were probed with anti-M13-HRP secondary antibody to detect bound phage. Shown is a single ELISA with three technical replicates; (B) Specificity of RTB-D1 or RTB-D2 capture ELISAs. Microtiter plates coated with indicted mAb were blocked with 2% (w/v) bovine serum albumin (BSA) in PBS then incubated with 10 μg/mL of ricin, RTA or RTB before being probed with 5 x 10^10^ PFU per well of RTB-D1 or RTB-D2. The plates were developed with anti-M13-HRP secondary antibody and TMB, as noted above. (JPG) [file pone.0236538.s001.jpg]
