# Supplementary material for: Sites of vulnerability on ricin B chain revealed through epitope mapping of toxin-neutralizing monoclonal antibodies
Source: PLoS One. 2020 Nov 9;15(11):e0236538. doi: 10.1371/journal.pone.0236538 (PMC7652295; doi:10.1371/journal.pone.0236538)
Supplement: S1 Table — (PDF) [file pone.0236538.s002.pdf]

**S1 Table. PCR primers used in this study**	
**PCR (5’-3’)**	
NotI-**RTB1**-F	ATAAGAATGCGGCCGCT**GCT**GATGTTAGTATGGACCCTGAGCCCATAG
NotI-**RTB136**-F	ATAAGAATGCGGCCGCT**AAT**ACACAACCTTTTGTGACAACCATTGTTGG
AscI-**RTB135**-R	TTGGCGCGCCCC**ATT**AGTAGGAAGCCAACCTTGACTAACGGCATA
AscI-**RTB262**-R	TTGGCGCGCCCC**AAA**TAATGGTAACCATATTTGGTTTGGGTCACCATGG
**Sequencing**	
JSC-HU	CACTTTATGCTTCCGGCTCG
JSC-rHU	TTGTCGTCTTTCCAGACG
Restriction sites are underlined. The codon that represents the first codon of RTB residue is **bold**. The base mutation that induces that C4S AA change in the first primer is double underlined.	
