# Supplementary material for: Sites of vulnerability on ricin B chain revealed through epitope mapping of toxin-neutralizing monoclonal antibodies
Source: PLoS One. 2020 Nov 9;15(11):e0236538. doi: 10.1371/journal.pone.0236538 (PMC7652295; doi:10.1371/journal.pone.0236538)
Supplement: S2 Table — (PDF) [file pone.0236538.s003.pdf]

S2 Table. Primers used for amplification of RTB domain

**Construct**	**Residues**	**Forward primer**	**Reverse primer**
RTB-FL	1-262	NotI-**RTB1**-F	AscI-**RTB262**-R
RTB-D1	1-135	NotI-**RTB1**-F	AscI-**RTB135**-R
RTB-D2	136-262	NotI-**RTB136**-F	AscI-**RTB262**-R
