# Supplementary material for: Sites of vulnerability on ricin B chain revealed through epitope mapping of toxin-neutralizing monoclonal antibodies
Source: PLoS One. 2020 Nov 9;15(11):e0236538. doi: 10.1371/journal.pone.0236538 (PMC7652295; doi:10.1371/journal.pone.0236538)
Supplement: S1 Text — (PDF) [file pone.0236538.s004.pdf]

Systematic Assignment of anti-RTB mAb Epitopes

The following text describes the mAb epitopes as presented in **Figure 4** of the manuscript. In that figure, ricin (PDB 2AAI) is shown in surface representation, with RTA colored light gray and RTB colored dark gray. The lactose molecules bound to the two carbohydrate recognition sites (CRDs) are colored brownish green (the 1α binding site) and teal (the 2γ binding site). We will rely on the information gained from this study (the domain of RTB that each mAb binds to, and the competition between mAbs and other mAbs, and between mAbs and VHHs) as well as information from previous studies (phage display epitope data, differential binding between RTB and a closely related toxin, Ricinus communis agglutinin I (RCA1)) and unpublished future manuscripts. Information from this study will cite the relevant figure in the text, while data from previous studies or unpublished data will be cited appropriately.

The most obvious place to start is with the three known mAb epitopes. The epitopes of the RTA mAbs SyH7 and JD4 were determined by hydrogen deuterium exchange, and are colored brown and cyan, respectively (Toth 2017). JB11 (previously published under the name "C/MA2") was found by phage display to bind primarily to a short linear stretch of amino acids 194-DSNIR-198 and secondarily to the degenerate linear stretch 54-DNTIR-58, both colored green (Yermakova 2012). By co-crystal structure (Rudolph MR, Vance DJ, Mantis NJ, manuscript in prep), the single domain antibody V2C11 (Vance 2017) binds to RTB directly in RTB's 2γ CRD (as shown by lactose colored in teal), more or less between the epitopes of JB11 and SyH7.

To place the epitopes or the remaining 9 anti-RTB mAbs, we will first consider LC5. It binds to domain 2 (**Figure 2B**) but does not compete with any of the cluster 6 mAbs (**Figure 2A**), so far represented on the epitope map by JB11, in green. Additionally, due to competition with JIZ-B7, which also competes with SyH7 (**Figure 3A**), LC5's epitope must be somewhat near the SyH7 epitope (brown) without also competing with SyH7 or JB11 (green). Thus, it is likely on the opposite side of domain 2 from JB11 and the rest of cluster 6. We therefore positioned LC5's epitope within RTB subdomains 2α and 2β, roughly corresponding to residues 167-185 (colored red). This span is 100% identical to RCA1, and indeed LC5 retains complete ability to recognize RCA1 (Rong 2017).

Next we will consider 24B11. 24B11 was initially described in 2006 (McGuinness 2006) and is the primary antibody with which competition defines membership in cluster 6. Like the

rest of cluster 6 it binds on Domain 2 (**Figure 2B**), but its competition profile against the VHHs is very different from the other mAbs in cluster 6 (**Figure 3A**). VHHs that compete with the RTA cluster 2 mAb SyH7 are termed "supercluster 2" VHHs, and the other 5 mAbs in cluster 6 compete with several of these supercluster 2 VHHs. However, 24B11 does not compete with any of the supercluster 2 VHHs, likely placing its epitope far away from the epitope of SyH7 on RTA. As well, VHHs V5C4 and V5B1 can compete with both LC5 and 24B11 (**Figure 3A**), placing the epitopes of those two mAbs somewhat close to one another. We therefore place 24B11 on the underside of RTB, in blue, roughly between JB11 and LC5, though closer to JB11 since 24B11 can compete with JB11 but not LC5 (**Figure 2A**).

The remaining cluster 6 mAbs 8A1, BJF9, MH3 and LF1 compete with the supercluster 2 VHHs to varying degrees, as well as their fellow cluster 6 mAb JB11, putting them near the SyH7 epitope (brown), the JB11 epitope (green) and the 2γ lectin site (lactose, teal) (**Figure 3A**). However, none of the cluster 6 mAbs compete with SyH7 at all, so they cannot be too close to SyH7's epitope (**Figure 2A**). Thus, we propose that these mAbs recognize overlapping epitopes on RTB subdomain 2β and 2γ, roughly spanning residues 190-250. This region of RTB shares a relatively high degree of identity with RCA1 (85%), and correspondingly the six MAbs display differential recognition of RCA1. While 24B11 and MH3 are fully reactive with RCA1, LF1 and 8A1 do not recognize RCA1 at all. BJF9 and JB11 recognize RCA1 to an intermediate degree (Rong 2017). JB11's known epitope (194-198), as described above, contains 2 mutations, including an R to K mutation in JB11's recognition sequence (DxNxR) (Yermakova 2012). This conservative mutation may explain why JB11 retains some binding to RCA1. 24B11, as described above, was placed on the bottom of RTB, and the primary sequence in this region (roughly 203-209) is 100% identical to RCA1, explaining 24B11's ability to bind RCA1. Given MH3's ability to bind RCA1, and its similar neutralizing activity as 24B11 (Rong 2017), we also placed MH3 in this region (dark red), though slightly higher on RTB (residues 211-214, also 100% identical to RCA1) due to its ability to compete with supercluster 2 VHHs, unlike 24B11 (**Figure 3A**).

LF1 (in orange) was next placed just above JB11's epitope. As noted above, LF1 does not recognize RCA1. The orange colored residues in **Figure 4** are residues 229-231 and 249. While only one of these four residues are mutated in RCA1 (S229N), residues 247-248 and 250 are mutated, which likely alters the conformation of residue 249. Note also that the colored

region is only a small estimate of the epitope, as coloring larger epitopes would have precluded showing approximate epitopes of all six cluster 6 mAbs. The actual epitope is likely much larger, potentially contacting the other mutated residues.

Finally, mAbs BJF9 and 8A1 are placed higher up on RTB still, tucked up against RTA. These epitope placements are supported by the fact that both BJF9 and 8A1 can partially compete with the RTA mAb JD4 (cyan), which binds to RTA just above the RTA/RTB interface (**Figure 2A**). They are further defined by their differential ability to bind to RCA1 (Rong 2017). As noted above, 8A1 does not bind to RCA1 at all, while BJF9 binds to an intermediate degree. Thus, 8A1's epitope (pink) is placed on residues 250-252, near the region of large differences with RCA1 described above for LF1. BJF9 is then placed on residues 218-225, (magenta) a region that is 100% identical to RCA1, but also spatially near the same regions of difference described for LF1 and 8A1.

With cluster 6 mAbs assigned, we next consider mAb 8B3. 8B3 competes with all of the cluster 6 mAbs and partially with the RTA mAb JD4 (**Figure 2A**), and also likely binds to the border between RTB's domain 1 and 2 (**Figure 2B**). Thus, the most likely location for its epitope to be located is on the domain 1-2 border directly adjacent to the cluster 6 mAbs, on the same side of RTB. Shown in purple, 8B3's epitope includes residues of RTB's subdomains 1γ and 2γ, stretching from residues 95 to 227. 8B3 has also been shown to retain almost complete recognition of RCA1 (Rong 2017), and in support of that finding, the residues on the surface in this region of RTB are almost entirely conserved.

We refer to mAb 8B3 as a cluster 5-6 mAb because it not only competes with all mAbs in cluster 6, but also with both mAbs in cluster 5, SylH3 and JB4 (**Figure 2A**). We also know that both SylH3 and JB4 capture domain 1 (**Figure 2B**), and that neither mAb can bind to RCA1 (Rong 2017, Yermakova 2012). Finally, SylH3 and, to a lesser degree, JB4, can compete with the RTA mAb JD4 (**Figure 2A**). In totality, this information significantly narrows down the potential epitope placement of these two mAbs. Thus, we can place the epitope for both of these mAbs on the loop regions of RTB's subdomain 1β to 1γ, corresponding to residues 65-105 (yellow). This region shares only 62.5% homology with RCA1, explaining the lack of binding of both mAbs with RCA1.

For now, we don't know what to make of JB4's apparent ability to also capture Domain 2 (**Figure 2B**). JB4 does not compete with any of the 7 mAbs that bind to domain 2 (BJF9, 8A1,

LF1, JB11, MH3, 24B11, and LC5), nor any of the 7 VHHs that bind to domain 2 (JIZ-B7, V5E4, V5G1, V5H2, V2C11, V2D4, V4A1). Combined, these 14 antibodies cover a significant portion of RTB's domain 2, and it is difficult to find a completely separate location where JB4 could bind to domain 2. Additionally, phenotypically JB4 behaves like SylH3 in every other assay, a result that would make sense given overlapping epitopes but much less sense if JB4 has two independent epitopes. We also considered the possibility that JB4 binds to the D1-D2 interface with sufficiently large surface area on each domain so as to be able to capture the domains independently. While this is a possibility, it still would be difficult to explain how JB4 does not complete with any of the domain 2 mAbs or VHHs. Additionally, the D1-D2 interface region is mostly conserved between RTB and RCA1 at the amino acid level, a fact inconsistent with JB4's inability to bind to RCA1.

Several of these mAbs, including JB4, are currently undergoing crystallization trials in an attempt to refine their epitopes in far greater detail.
