# Supplementary material for: Sites of vulnerability on ricin B chain revealed through epitope mapping of toxin-neutralizing monoclonal antibodies
Source: PLoS One. 2020 Nov 9;15(11):e0236538. doi: 10.1371/journal.pone.0236538 (PMC7652295; doi:10.1371/journal.pone.0236538)
Supplement: Attachment — Submitted filename: R2R PONE-D-20-21014-Final.pdf [file pone.0236538.s005.pdf]

Response to Reviewers [PONE-D-20-21014]

Reviewer #1: Following this review the recommendation is accept without revisions.
Response: We thank Reviewer 1 for his/her favorable review of the manuscript.

Reviewer #2: Overall, this study proposes to re-define the epitopes of two well characterized and mechanistically distinct neutralizing mAbs further informing the location of Ricin B protective epitopes. While important, these conclusions contradict prior work yet rely upon a single experimental approach using phage-displayed proteins which were not characterized for proper folding. If the authors propose changing previously described epitope maps, it is important to include two complementary experimental approaches with proper control data that support the same new conclusion.

Response: The Reviewer makes a valid point in noting the importance of a secondary methodology to confirm/validate the proposed epitope locations describe in this manuscript. The most compelling evidence in this respect are the X-ray co-crystal structures of SylH3 and V5E4 in complex with ricin holotoxin. The two recently refined structures confirm SylH3 and V5E4's proposed epitope location, as shown in Figure 4. There are two separate manuscripts nearing submission that will describe these structures. To use these structures as a means to validate the current study, we will link the current manuscript to those future manuscripts using "Crossref," which is offered as part of PLOS publications. Of course, the structures will also be available on the PDB.

1. The data would be more compelling if at least some of the data are shown with the antibody dilution curve to allow readers to evaluate the dynamic range of the assays and visualize the degree of competition used to derive the +, ++ and +++ metrics shown in the tables. I am surprised that the phage ELISAs were performed with one phage concentration, 5x10^10 pfu per well and did not serially dilute the phage to (a) visualize the dose-response and (b) use these data to rank clones by relative affinity as a secondary check on data consistency with other methods.

Response: As requested by the Reviewer, we have now included **S1 Figure** showing a dose-response curve of SylH3 and 24B11 capturing RTB-D1 and RTB-D2, respectively. The data demonstrate that 5x10^10 PFU is within the dynamic range of the dose-response curve. Moreover, also included in that figure are competition assays with ricin, RTB and RTA to demonstrate specificity of the RTB-D1 and RTB-D2 phage.

2. I am perplexed by the statement on lines 100-103 that there is a "proven inability to express recombinant RTB in E. coli" however, "full-length RTB as well as RTB-D1 and D2 constructs have been successfully expressed as fusion proteins on the tip of filamentous phage M13." These statements seem contradictory since M13 phage fusion proteins are also produce recombinantly in E. coli and exported from the cytoplasm to the periplasm by Sec machinery before phage assembly. What tests have been performed to assess the structural

and functional integrity of the phage displayed RTB and how does recombinantly expressed soluble RTB perform on these same tests? Recombinant RTB produced in E. coli is commercially available, which suggests (but does not guarantee) that it is functional. The rational for using phage displayed RTB should use more robust logic and controls to determine whether (or the extent to which) the displayed proteins are properly folded and active shown.

Response: While the Reviewer is correct in that RTB has been expressed in *E.coli* when targeted to the periplasmic space, the overall yield of properly folded protein is low and highly strain dependent {Hussain, 1989; Wales, 1994}. Moreover, mutagenesis of RTB has only been done in eukaryotic expression systems such as Xenopus oocytes (Newton, 1992). However, as reported by Swimmer (1982), full length and individual domains of RTB are amenable to M13 display following expression (and secretion) by *E.coli*. Considering our lab's expertise in M13-phage display, we chose to that as the basis for the current study.

To address the very astute comment regarding "…. tests have been performed to assess the structural and functional integrity of the phage displayed RTB and how does recombinantly expressed soluble RTB perform on these same tests?" we now provide competition assays with native ricin and RTB (**S1 Figure**).

Minor comments

1. Line 156: single domain Vhh ELISas were performed. Response: Corrected

2. Add a sentence of the VHH construct (i.e. includes E-tag which is then used for detection in ELISAS). Response: As requested, we have modified the text to state "The V_HHs used in this study carry a C-terminus E epitope tag (E-tag; GAPVPYPDPLEPR) for the purpose of detection by ELISA using HRP-conjugated, affinity-purified anti-E-tag rabbit IgG.

3. Tables 1 and 2 define FL (Full length). Response: Corrected

Reviewer #3: My only 'criticism' would be that it is highly incremental relative to the author's previous work in the area. Having said that, the paper gets results from an analytically challenging system and corrects an earlier (less confident) report by the same authors that misidentified the epitope for one of the two antibodies, and for this, the authors should be commended.

Response: We thank Reviewer 3 for his/her favorable review of the manuscript.
